# Distinct Mechanisms of Biotic and Chemical Elicitors Enable Additive Elicitation of the Anticancer Phytoalexin Glyceollin I

**DOI:** 10.3390/molecules22081261

**Published:** 2017-07-27

**Authors:** Kelli Farrell, Md Asraful Jahan, Nik Kovinich

**Affiliations:** 1 Department of Biology, West Virginia University, Morgantown, WV 26506, USA; kcfarrell@mix.wvu.edu; 2 Division of Plant and Soil Sciences, West Virginia University, Morgantown, WV 26506, USA; mjahan@mix.wvu.edu

**Keywords:** bioproduction, phytoalexin, isoflavonoid, glyceollin, soybean [*Glycine max* (L.) Merr.]

## Abstract

Phytoalexins are metabolites biosynthesized in plants in response to pathogen, environmental, and chemical stresses that often have potent bioactivities, rendering them promising for use as therapeutics or scaffolds for pharmaceutical development. Glyceollin I is an isoflavonoid phytoalexin from soybean that exhibits potent anticancer activities and is not economical to synthesize. Here, we tested a range of source tissues from soybean, in addition to chemical and biotic elicitors, to understand how to enhance the bioproduction of glyceollin I. Combining the inorganic chemical silver nitrate (AgNO_3_) with the wall glucan elicitor (WGE) from the soybean pathogen *Phytophthora sojae* had an additive effect on the elicitation of soybean seeds, resulting in a yield of up to 745.1 µg gt^−1^ glyceollin I. The additive elicitation suggested that the biotic and chemical elicitors acted largely by separate mechanisms. WGE caused a major accumulation of phytoalexin gene transcripts, whereas AgNO_3_ inhibited and enhanced the degradation of glyceollin I and 6″-*O*-malonyldaidzin, respectively.

## 1. Introduction

Plants, like other organisms, have metabolic pathways that remain silent until activated by stresses. Phytoalexins are defense metabolites biosynthesized in response to pathogens, but which for unknown reasons also accumulate in response to specific environmental stresses and inorganic chemicals, such as heavy metals [1,2,3]. Much of what is known about phytoalexin elicitation mechanisms comes from studies of the glyceollins in soybean, camalexins in Arabidopsis, diterpenoids and flavonoids in rice, stilbenes in grapevine, alkaloids in California poppy, and the 3-deoxyanthocyanidins, terpenoids, and phytodienoic acids in maize. However, very few studies have attempted to distinguish the elicitation mechanisms of biotic and chemical elicitors.

Biotic elicitation begins when a microbial derived pathogen-associated molecular pattern (PAMP) or effector binds to a pattern recognition receptor at the plasma membrane of the plant cell. Mitogen-activated protein kinase (MAPK) or phospholipase signaling ultimately results in the expression of transcription factors (TFs) that directly activate the transcription of phytoalexin biosynthesis genes. MYB-, bHLH-, or WRKY-type TFs directly activate some or all of the phytoalexin biosynthesis genes in cotton, sorghum, rice, Arabidopsis, and grapevine [4,5,6]. In soybean, no phytoalexin TF has been identified, but transcription of glyceollin biosynthesis genes was coordinately induced in response to the pathogen *Phytophthora sojae* [7,8].

Heavy metals, such as silver nitrate (AgNO_3_), have elicited chemically diverse phytoalexins in many plant species. The molecular target(s) of these heavy metals remain(s) unknown. AgNO_3_ was shown to inhibit developmental processes triggered by exogenous ethylene treatment, and thus has been considered a potent inhibitor of ethylene perception [9]. Some evidence has suggested that AgNO_3_ and pathogens elicit phytoalexins primarily by different mechanisms. The *P. sojae*-resistant soybean variety Harosoy 63 elicited glyceollins rapidly in response to race 1 *P. sojae*, but the susceptible variety Harosoy did not, whereas both varieties responded similarly to AgNO_3_ [10]. Feeding AgNO_3_-elicited soybean cotyledons the radiolabeled intermediate phenylalanine did not result in radiolabeled glyceollins, but AgNO_3_ treatment reduced the degradation of radiolabeled glyceollins [11].

**Figure 1:**
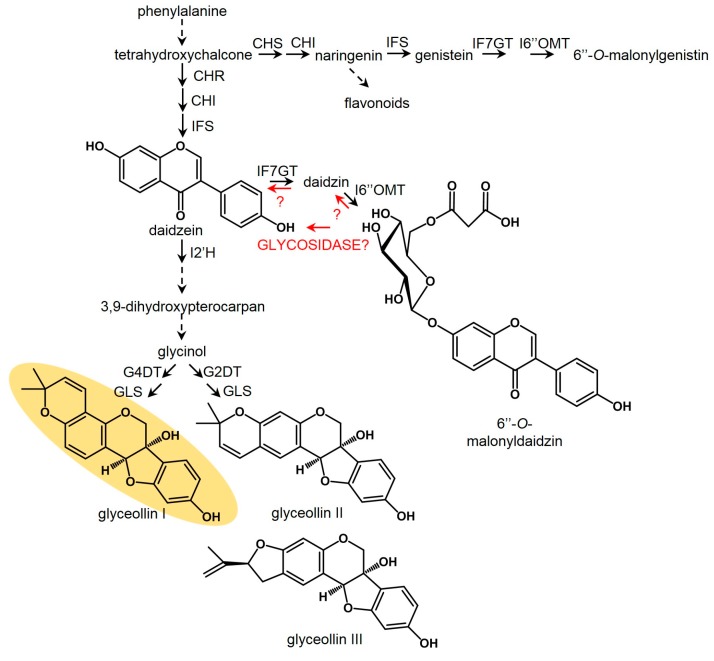
Glyceollin I biosynthetic pathway. In addition to de novo biosynthesis, the constitutively accumulating isoflavone conjugate 6″-*O*-malonyldaidzin may be hydrolyzed to provide daidzein intermediates for glyceollin I biosynthesis. CHS, chalcone synthase; CHR, chalcone reductase; CHI, chalcone isomerase; IFS, isoflavone synthase; I2′H, isoflavone 2′-hydroxylase; G4DT, glycinol 4-dimethylallyl transferase; G2DT, glycinol 2-dimethylallyl transferase; GLS, glyceollin synthase; UF7GT (UGT88E3) UDP-glucose:isoflavone-7-*O*-glucosyltransferase; I6″OMT (GmMT7) isoflavone-7-*O*-glucoside-6″-*O*-methyltransferase.

Glyceollins are the major phytoalexins of the soybean. They belong to the pterocarpan subclass of isoflavonoids, which possesses great potential as scaffolds for pharmaceutical development [12]. Glyceollins are biosynthesized from the isoflavonoid daidzein, which can result from de novo biosynthesis beginning at phenylalanine, or possibly from the hydrolysis of preformed isoflavonoid-glycoside conjugates (Figure 1). Glyceollins have broad-spectrum antiproliferative or antitumor activities against human lung, breast, prostate, ovary, skin, and kidney cancers. Glyceollin I is the most potent, and directly antagonizes human estrogen receptor α (ERα) and ERβ [13]. Glyceollin I also exhibits a rare ER-independent mode-of-action via a mechanism that is not yet fully understood [14]. In light of the therapeutic potential of glyceollin I, studies have attempted to produce large-scale (gram) amounts by chemical synthesis or by the elicitation of soybean [15,16]. However, the yield by chemical synthesis was up to 12%, and required a highly concerted effort of specialists six months to complete, rendering it uneconomical for commercial production [15]. Here, we aimed to identify which soybean tissues and treatments provide optimal glyceollin I bioproduction in vitro. Our study provides novel insight into how biotic and chemical elicitation pathways are distinct, and how this can be exploited to enhance the bioproduction of glyceollin I.

## 2. Results and Discussion

### 2.1. Imbibing Soybean Seeds Are the Most Abundant Source of Glyceollin I

Prior studies have found that glyceollins were readily elicited up to the first-true-leaf stage of development, when they were essential for defense against *P. sojae* [10,17,18]. They were also elicited in transgenic hairy roots and soybean seeds [19,20,21]. Yet, no single study has compared different soybean tissues to determine which produces the most glyceollin I.

We compared the organs of seedlings at the first-true-leaf stage, imbibing seeds, and hairy roots. The samples were treated with the well-characterized wall glucan elicitor (WGE) isolated from the mycelium of *P. sojae* [22]. The organs of the seedlings and imbibing seeds were spot-treated on a wound of equivalent size with the same amount of WGE, and tissue of roughly the same mass encompassing the wound site was collected for the measurement of glyceollin I (see Methods).

**Figure 2:**
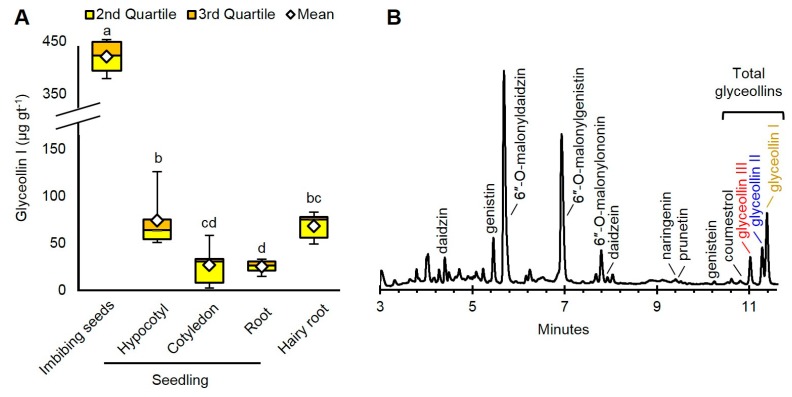
(**A**) Amounts of glyceollin I from soybean organs treated with wall glucan elicitor (WGE) from *Phytophthora sojae*. Two-way ANOVA, Tukey post hoc test (*p* < 0.001); (**B**) UPLC-PDA chromatogram at 283 nm of isoflavonoids from imbibing seeds. Isoflavonoid identities were determined by UPLC-PDA-MS^n^ retention time, fragmentation pattern, and absorbance features by comparison to standards (commercial or purified) and by comparison to the literature (Supplementary Table S1). Different letters show significant differences by ANOVA.

The imbibing seeds produced the highest amount of glyceollin I: 421.7 µg per gram fresh tissue (gt^−1^) (Figure 2A). This amount was roughly sixfold greater than that of hairy roots and hypocotyls, and 16-fold greater than cotyledons and roots. The glyceollin I from the imbibing seeds represented a major peak at 283 nm absorbance (Figure 2B).

Since glyceollin I is costly to purify from other glyceollins [23], we determined which tissue had the greatest purity of glyceollin I relative to the other glyceollins. The roots produced 85.9% glyceollin I (Supplementary Figure S1), but produced the lowest total amount with our elicitation parameters (Figure 2A). Since imbibing seeds produced the greatest total amounts of glyceollin I and exhibited the second greatest purity (68.5%), we decided to focus on imbibing seeds to compare the biotic and chemical elicitation mechanisms of glyceollin I.

### 2.2. Wall Glucan Elicitors from P. sojae and Pythium Elicit More Glyceollin I Than Rhizopus, Aspergillus, and Fusarium Microspores at Standard Treatment Concentrations

A wide variety of fungal, nematode, and oomycete pathogens elicit glyceollins. The commonly studied microspore elicitors are from the *Rhizopus* and *Aspergillus* species, whereas WGE is most commonly from *P. sojae* [24,25,26]. To compare the elicitation features of several biotic elicitors at typical treatment concentrations, we treated imbibing soybean seeds with 7 × 10^7^ CFU mL^−1^ microspores from *Aspergillus fumigatus*, *Rhizopus nigricans*, and *Fusarium tricinctum*, or WGEs from *P. sojae* and *Pythium* at 20 mg mL^−1^.

**Figure 3:**
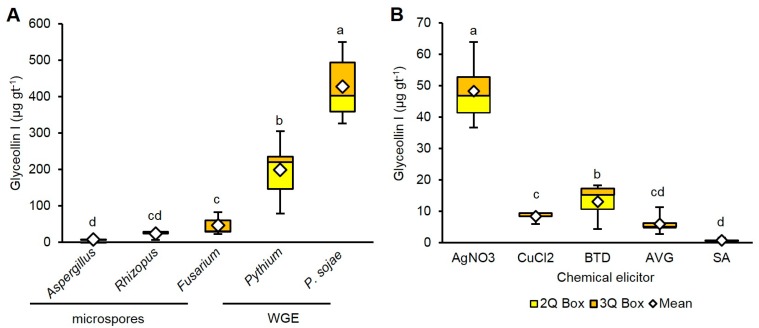
(**A**) Amounts of glyceollin I from soybean seeds treated with biotic elicitors for 24 h. Two-way ANOVA, Tukey post hoc test (*p* < 0.001); (**B**) Treatment of seeds with chemical elicitors (AgNO_3_), copper chloride (CuCl_2_), benzothiadiazole (BTD), aminoethoxyvinyl glycine (AVG), and salicylic acid (SA) at 1 mM. Different letters show significant differences by ANOVA.

WGE from *P. sojae* induced the highest amounts of glyceollin I (428.3 µg gt^−1^), followed by *Pythium* (199.0 µg gt^−1^) (Figure 3A). Glyceollin I comprised more than 50% of the total glyceollin amounts elicited by *P. sojae* and *Pythium*, whereas it was less than 50% for the fungal microspore elicitors (Supplementary Figure S1).

### 2.3. AgNO_3_ Elicits More Glyceollin I Than CuCl_2_, BTD, AVG, and SA at Equivalent Treatment Concentrations

Chemical elicitors, such as heavy metals or inorganic compounds, that stimulate or inhibit components of the plant immune system can function as phytoalexin elicitors. We chose to compare under the same conditions as our biotic elicitors the elicitation potential of the heavy metals AgNO_3_ and CuCl_2_ and inorganic compounds that affect the immune system. The duration of elicitation for our experiments was initially set to 24 h, since that is the duration typically reported in previous studies. Benzothiadiazole (BTD) stimulated the immune system by functioning as a mimic of the plant hormone salicylic acid (SA) [27]. Aminoethoxyvinyl glycine (AVG) inhibited the biosynthesis of ethylene [28], and thus could be compared to the putative inhibitor of ethylene perception, AgNO_3_.

At 1 mM treatment concentration, AgNO_3_ was the most potent and elicited 48.2 µg gt^−1^ glyceollin I (Figure 3B). That was eightfold more than AVG. BTD was the second most potent, eliciting 48.2 µg gt^−1^, which was 17-fold more than SA. Only AgNO_3_ preferentially elicited glyceollin I (Supplementary Figure S1).

### 2.4. AgNO_3_ and P. sojae WGE Elicit Glyceollin I with Different Dynamics

Since AgNO_3_ and WGE from *P. sojae* were the most potent chemical and biotic elicitors at the treatment concentrations tested, we chose to investigate in more detail their mechanisms, beginning with elicitation dynamics. AgNO_3_ elicited the accumulation of glyceollin I in a biphasic fashion, with peaks at 24 h and 96 h after treatment (Supplementary Figure S2). By contrast, WGE reached a maximum at 24 h that was sustained. To distinguish effect of each elicitor from that of the wounding, we calculated the fold change in metabolite levels of each elicitor relative to the water control. WGE caused the greatest induction over the wounded control at 48 h, whereas AgNO_3_ was at 72 h (Supplementary Figure S2).

### 2.5. P. sojae WGE and AgNO_3_ Elicit the Accumulation of Glyceollin I Mainly by Distinct Mechanisms

Since the elicitation dynamics were different for WGE compared to AgNO_3_, we anticipated that each elicitor functioned by a different mechanism. We tested this hypothesis by first identifying the optimal concentration of each elicitor required to elicit the maximum accumulation of glyceollin I, and then combined the elicitors at these maximum concentrations. The rationale was that the combined treatment would result in a greater accumulation of glyceollin I only if the elicitation mechanisms were different. To determine the maximum level of glyceollin I that WGE and AgNO_3_ can respectively elicit, we conducted dose response analyses. Germinating soybean seeds were treated with up to 10 mM AgNO_3_ and 60 mg mL^−1^ WGE.

**Figure 4:**
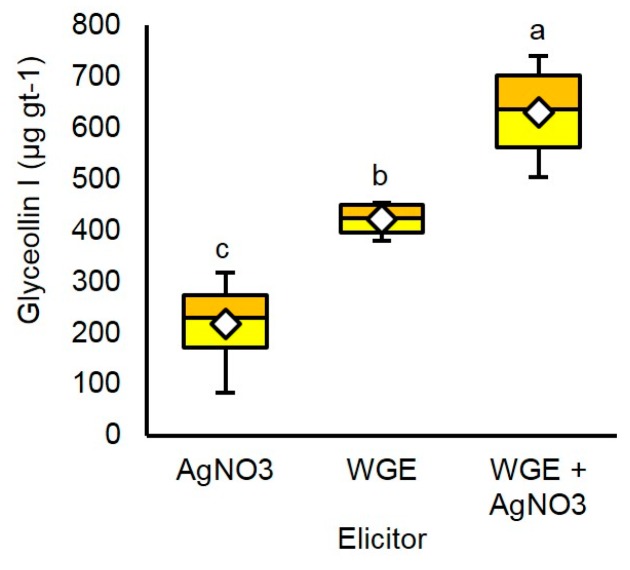
Elicitation with AgNO_3_ (5 mM) and WGE (20 mg mL^−1^) separately and in combination. Two-way ANOVA, Tukey post hoc test, *p* < 0.001. Different letters show significant differences by ANOVA.

At 48 h after treatment, 5 mM AgNO_3_ elicited a maximum mean concentration of glyceollin I of 202.5 µg gt^−1^ (Supplementary Figure S3). By contrast, 20 mg mL^−1^ WGE elicited more than double this amount, with a mean concentration of 449.8 µg gt^−1^. The combined treatment elicited a mean glyceollin I concentration of 635.8 µg gt^−1^ and a maximum of 745.1 µg gt^−1^ (Figure 4). This mean concentration was significantly greater than that observed for the single treatments, and was very close empirically to the sum of the individual elicitor treatments (640.0 µg gt^−1^). This strongly suggests that WGE and AgNO_3_ function by distinct elicitation mechanisms.

The combined treatment also enhanced the purity of glyceollin I to 89.4% of the total glyceollins and to 34.6% of the total seed isoflavonoids (Supplementary Figures S1 and S4, respectively).

### 2.6. P. sojae WGE and not AgNO_3_ Induces Major Accumulation of Glyceollin Gene Transcripts

Since glyceollin biosynthesis genes were regulated at the level of transcription in response to *P. sojae* [7,8,29], we determined whether the elicitors WGE and AgNO_3_ induced glyceollin biosynthesis at the mRNA level. We measured mRNA accumulation for genes spanning from general isoflavonoid to late-stage glyceollin I biosynthesis. The homologous genes *IFS1* and *IFS2* encode isoflavone synthase (IFS) enzymes that catalyze the first committed step in isoflavonoid biosynthesis (Figure 1). The enzyme isoflavone 2′-hydroxylase encoded by *I2′H* and the glycinol 4-dimethylallyltransferase encoded by *G4DT* catalyze the first committed steps for the biosynthesis of all glyceollins and glyceollin I, respectively [7,30].

**Figure 5:**
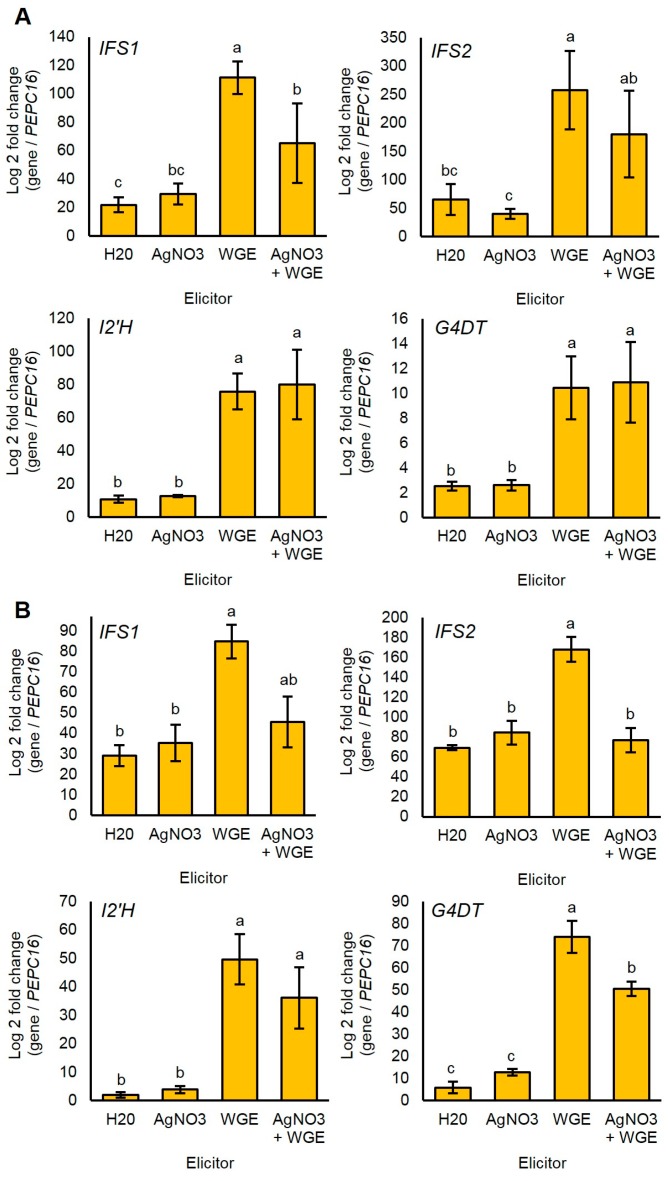
(**A**) Relative gene expression levels 48 h after elicitor treatment with elicitor measured by qRT-PCR; (**B**) Relative gene expression levels at 8 h after elicitor treatment. Expressions in each sample were measured relative to the endogenous reference gene *PEPC16*. Two-way ANOVA, Tukey post hoc test, *p* < 0.01. Two independent experiments with four biological replicates were conducted with similar results. Different letters show significant differences by ANOVA.

WGE induced the accumulation of the mRNAs of all four genes compared to the solvent control at 48 h after elicitation (Figure 5A). By contrast, AgNO_3_ did not significantly induce any genes at this time point. Further, the combined treatment did not have elevated levels of mRNAs compared to the WGE treatment, and even exhibited a reduced expression of IFS1. These results suggest that AgNO_3_ did not elicit glyceollins by stimulating the accumulation of glyceollin biosynthesis gene mRNAs.

Since the elicitation of phytoalexins is a transient process subject to feedback mechanisms, we also tested whether AgNO_3_ elicited the accumulation of biosynthesis gene mRNAs more rapidly than WGE. To test this, we measured mRNA levels at 8 h after treatment, a time point that preceded the measurable accumulation of glyceollin I. Again, AgNO_3_ did not elicit the accumulation of any of the mRNAs (Figure 5B). Further, the combined treatment exhibited less expressions of *IFS2* and *G4DT* compared to the WGE treatment, suggesting that the elevated levels of glyceollin I observed at 48 h were not due to regulation of biosynthesis at the mRNA level.

### 2.7. AgNO_3_ Inhibits the Degradation of Glyceollin I and Enhances the Specific Hydrolysis of 6″-O-Malonyldaidzin

Our gene expression measurements demonstrated that AgNO_3_ did not induce the expression of glyceollin biosynthesis genes. This suggested that AgNO_3_ acted via another mechanism to elicit glyceollin I in the soybean seed. Heavy metal elicitors have been suggested to elicit isoflavonoid phytoalexins by inhibiting their degradation or by increasing the rates of the hydrolysis of isoflavone conjugates to provide metabolic intermediates for phytoalexin biosynthesis [11,31]. To investigate whether AgNO_3_ inhibited the degradation of glyceollin I, we incubated dissected seeds in water or 5 mM AgNO_3_ for 2 h, then transferred them to an extract containing partially purified glyceollin I.

**Figure 6:**
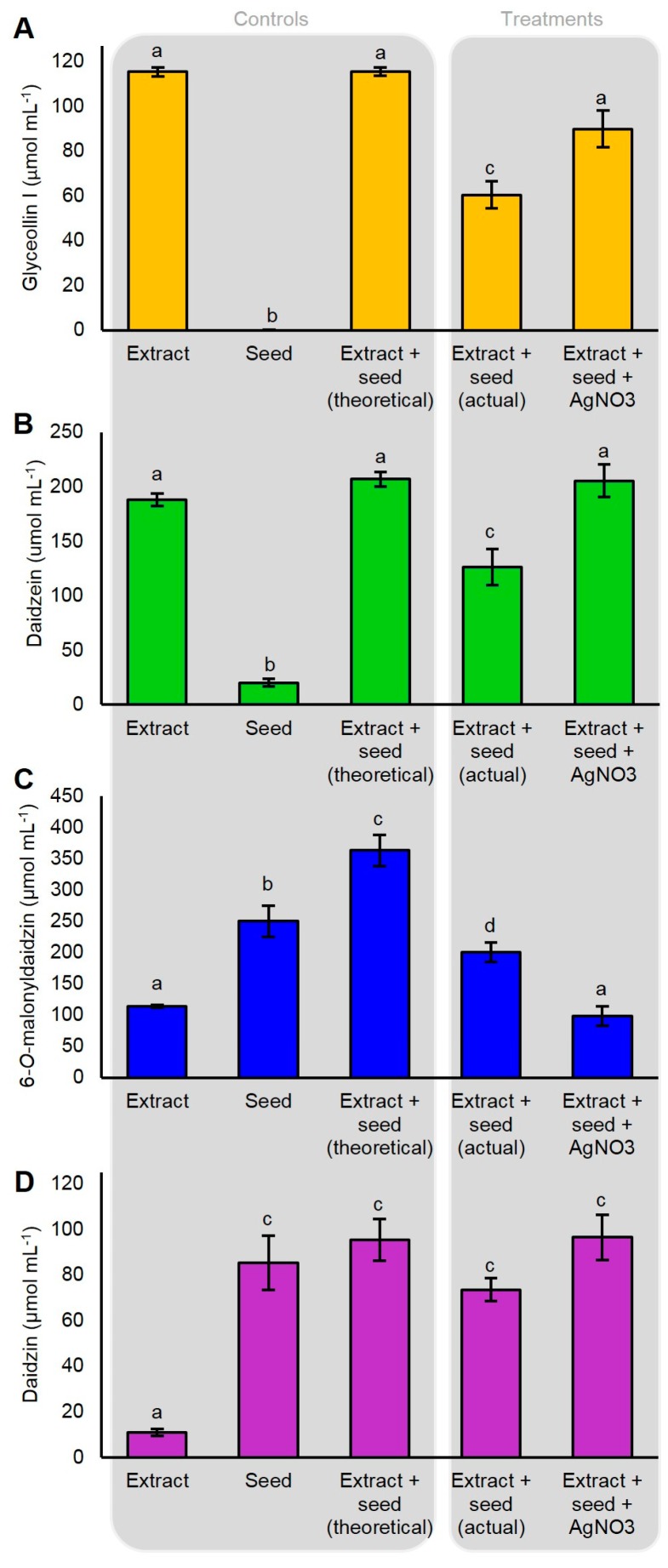
Degradation of externally supplied glyceollin I (**A**); daidzein (**B**); 6″-*O*-malonyldaidzin (**C**); and daidzin (**D**) by soybean seeds. Isoflavonoids were partially purified from an ethanolic extract of WGE-elicited soybean seeds, and were incubated with imbibed soybean seeds pretreated with AgNO_3_ or water to test for effects on metabolite degradation. Shown is the amount of metabolite from the initial partially purified ethanolic extract (Extract), the amount from imbibed seeds in incubated in water only (Seed), the theoretical amount from empirically adding the values from Extract and Seed (theoretical), the amount observed from incubating the seed in water for 2 h followed by metabolite extract for 4 h (actual), or from incubating the seed in 5 mM AgNO_3_ for 2 h followed by metabolite extract for 4 h (Extract + seed + AgNO_3_). Two-way ANOVA, Tukey post hoc test (*p* < 0.001). The results represent four biological replicates. Different letters show significant differences by ANOVA.

The incubation of the dissected seed with 115 umol mL^−1^ of glyceollin I resulted in the degradation of 54.9 µmol mL^−1^ (Figure 6A). The pretreatment of the seed with AgNO_3_ for 2 h did not cause a detectable induction of glyceollin I (not shown) and reduced this degradation by 25.7%, rendering the final amount statistically equivalent to the initial extract. A similar inhibition of degradation was observed for the intermediate daidzein (Figure 6B). By contrast, AgNO_3_ accelerated the hydrolysis of the isoflavone conjugate 6″-*O*-malonyldaidzin (Figure 6C). The levels of daidzin were unchanged compared to the control (Figure 6D). AgNO_3_ did not affect the levels of any of the metabolites in the absence of the seed (not shown), indicating that the changes in metabolite levels were catalyzed by seed enzymes. During the course of the experiment, no de novo biosynthesis of glyceollin I was detected from seeds incubated in the absence of the glyceollin I extract (Figure 6A). In summary, these results show that AgNO_3_ acts by inhibiting the degradation of glyceollin I and by accelerating the hydrolysis of 6″-*O*-malonyldaidzin, possibly to provide daidzein intermediates for glyceollin I biosynthesis.

### 2.8. Discussion

Previous attempts were made to biosynthesize and synthesize glyceollin I on a preparative scale. The most effective approach yielded 12% glyceollin I, but its scale up to produce gram amounts required ~6 months and a highly concerted team effort of highly experienced chemists [15]. A creative approach to biosynthesize prenylated isoflavonoids on a preparative scale involved the malting (as in the brewing industry) and simultaneous elicitation of 4.0 kg of germinating soybeans with the fungus *Rhizopus microsporus* [16]. This yielded up to 335 µg gt^−1^ glyceollin I in nine days. Thus, the overall yield from 4.0 kg of seeds could be 1.34 g in a fraction of the time needed for synthesis. Here, we demonstrated on an analytical scale that combining biotic and chemical elicitors resulted in an additive effect on the accumulation of glyceollin I, with WGE and AgNO_3_ eliciting almost double that amount (635.8 µg gt^−1^) in 48 h (Figure 4). The approach was labor-intensive and could benefit from mechanization, but if amenable to direct scale up would yield almost double the amount (2.54 g) of glyceollin I from 4.0 kg of seeds in 2–3 days.

Several lines of evidence suggested that this occurred because the chemical and biotic elicitors functioned by distinct mechanisms. Firstly, AgNO_3_ and WGE elicited different patterns of glyceollin I accumulation over time (Supplementary Figure S2). Second, our gene expression experiments demonstrated that AgNO_3_ did not increase the accumulation of essential glyceollin genes, whereas WGE from *P. sojae* highly upregulated both the isoflavonoid and glyceollin genes (Figure 5). Finally, AgNO_3_ inhibited the degradation of glyceollin I (Figure 6), whereas WGE did not [11]. These results suggested distinct genetic targets for enhancing biotic and chemical elicitation pathways by genetic engineering. Transcription factors (TFs) of the MYB, WRKY, and bHLH families directly activated the transcription of phytoalexin biosynthesis gene promoters, and thus their overexpression could be used to enhance the biotic elicitation pathway. Yet to our knowledge, TFs that activate specifically the heavy metal elicitation pathway have not been identified.

AgNO_3_ was shown to be a potent inhibitor of ethylene perception [9]. Yet, even though we applied the ethylene biosynthesis inhibitor AVG at four times the concentration found to fully inhibit ethylene biosynthesis in soybean [28], we observed 8.1-fold less elicitation compared to AgNO_3_. This suggested that the mechanism-of-action of AgNO_3_ on glyceollin I was mostly distinct from the inhibition of ethylene biosynthesis. AgNO_3_ and several other heavy metals inhibited the degradation of glyceollins in soybean cotyledons, suggesting that heavy metal elicitors may target a glyceollin degrading enzyme or pathway [11]. Our results confirmed this, but we also found that AgNO_3_ enhanced the degradation of the major isoflavone conjugate, 6″-*O*-malonyldaidzin, and reduced the consumption of daidzein (Figure 6). This suggested that AgNO_3_ acted by stimulating the expression or activity of an enzyme that catalyzed the hydrolysis of 6″-*O*-malonyldaidzin to provide daidzein intermediates for the biosynthesis of glyceollin I, rather than by solely inhibiting glyceollin degradation. Future studies should address whether AgNO_3_ directly enhances the activity of a glycosidase that hydrolyzes 6″-*O*-malonyldaidzin, or whether AgNO_3_ stimulates the upregulation of the corresponding glycosidase gene(s). Our results suggest that overexpressing a glycosidase with specificity for 6″-*O*-malonyldaidzin would enhance the heavy metal elicitation pathway. Glycosidase activities towards malonyl conjugates of daidzin and genistin were detected in the ƒseed and root exudates of soybean [32]. The jasmonic acid (JA)-inducible glycosidase G2 from *Medicago truncatula* has high specificity for daidzin (i.e., daidzein-7-*O*-glucoside) when expressed in yeast [31], but 6″-*O*-malonyldaidzin was not tested as a substrate. The significance of AgNO_3_ preferentially stimulating the degradation of 6″-*O*-malonyldaidzin and not daidzin remains unclear. Studies may have to address the subcellular localization of these metabolites relative to the glycosidase(s) capable of hydrolyzing them to understand this mechanism.

## 3. Experimental Section

### 3.1. Chemicals

Stocks of the chemical elicitors AgNO_3_ (Sargent Welch, Buffalo, NY, USA), CuCl_2_, and 2,1,3-benzothiadiazole were 5 M in water. Salicylic acid was 5 M in DMSO. Elicitors were purchased from Sigma. The glyceollin I synthetic standard was from Paul Erhardt (University of Toledo, Bancroft, MI, USA), daidzein from Cayman Chemical and daidzin, genistin (Ann Arbor, MI, USA), and genistein were from Indofine (Hillsborough, CA, USA). All UPLC solvents were LC-MS grade from Fisher (Hampton, FL, USA).

### 3.2. Plant Growth and Elicitation

Harosoy 63 seeds were sterilized in 70% ethanol 0.2% triton X (*v*/*v*) for 5 min, rinsed thrice with 95% ethanol, and imbibed in sterile water overnight. The imbibate was then discarded to remove growth inhibitors. For the elicitation of seeds, the seed coat was carefully removed to avoid damaging the embryo. The distal 2–3 mm of the cotyledons (the tips) were excised, and an incision was made through the axis of the central vein two thirds of the way towards the radical. Elicitor was applied to the wound, and the embryo was placed upright on its distal end on sterile wetted filter paper in a petri dish. For the elicitation of seedlings, sterilized seeds were transferred to water-soaked sterile vermiculite in beakers. The beakers were covered with a sterile cheese that had a circular hole cut into it to permit the passage of light. The cloth was covered with plastic wrap to ensure aseptic growth. The seed coats were carefully removed within 1–2 days. Seedlings were grown until the first-true-leaf stage (~1 week). For elicitation, a knotch ~3 mm in diameter and 1–2 mm deep was cut into the central vein on the abaxial side of the cotyledon or into the hypocotyl, and roughly 250 mg tissues (the weight of a seed) were harvested for the measurement of glyceollin I. Hairy root transformation with *Agrobacterium rhizogenes* strain K599 was done according to [33]. The roots and hairy roots were elicited by cutting ~250 mg into 1 cm pieces and applying elicitor over the wounds. All seedling growth and elicitation treatments were done under 24 h cool white T5 fluorescent lights (500 μE m^−2^ s^−1^) at 24 °C. The samples were treated with 50 µL of elicitor for 48 h unless otherwise specified. The hairy roots and roots were treated for 24 h. WGE was purified from race 1 *P. sojae* according to [22], with the exception that suction filtration was replaced by centrifugation at all steps.

### 3.3. Isoflavonoid Analyses

For the extraction of isoflavonoids, elicited seeds were pulverized for 3 min using 5 mm stainless steel grinding balls in a MM400 mixer mill (Retsch, Newtown, CT, USA) at frequency of 30/s, then again following the addition of 80% ethanol (1 µL mg^−1^ fresh tissue). Seedling organs were ground using a mortar and pestle following freeze-drying, and were extracted similarly to seeds. The samples were centrifuged at 17,000× *g* for 3 min, clarified at −20 °C overnight, then centrifuged again. The supernatant was filtered through a 0.2 µm, and 1 µL was analyzed by UPLC-PDA-MS^n^ adapted from [24]. The quantity of each isoflavonoid was determined by comparison of the area under their peak at 283 nm absorbance in comparison to a standard curve of authentic standard. To estimate the amount of glyceollin I relative to all other seed isoflavonoids, unknown compounds and compounds for which we did not have standards were quantified in comparison a standard curve of daidzin.

### 3.4. UPLC-PDA-MS^n^

UPLC-PDA-MS^n^ was conducted using an Accela system (Thermo Scientific, San Jose, CA, USA) consisting of a 1250 pump, Open AS autosampler, and photodiode array (PDA) detector connected to a Q-Exactive–Oribitrap MS containing a HESI. Separations were achieved with an Acquity UPLC BEH shield RP18 column (2.1 mm i.d. × 150 mm, 1.7 μm particle size; Waters, Milford, MA, USA) with an Acquity UPLC BEH shield RP18 VanGuard precolumn (2.1 mm i.d. × 5 mm, 1.7 μm particle size; Waters). The solvents, at a flow rate of 300 μL/min, were water acidified with 0.1% (*v*/*v*) acetic acid, eluent A, and ACN acidified with 0.1% (*v*/*v*) acetic acid, eluent B. The temperature of the column oven was 35 °C. The elution profile was as follows: 0−2 min, from 10% to 25% (*v*/*v*) B; 2−9 min, from 25% to 50% B; 9−12 min, 50% B; 12−17 min, from 50% to 100% B; 22−24 min, 100% to 10% B; and 24−30 min, 10% B. MS analysis was performed in Full MS/AIF mode in both positive and negative polarities. The Full MS properties were: Resolution 70,000, AGC target 3e6, Maximum IT 200 ms, and Scan range 120−1200 *m*/*z*. AIF properties were: Resolution 70,000, AGC target 3e6, Maximum IT 200 ms, NCE 35, and Scan range 80−1200 *m*/*z*. Nitrogen was used as sheath and auxiliary gas.

### 3.5. qRT-PCR

For the gene expression measurements, seeds were harvested into liquid nitrogen and lyophilized for at least 3 days. Tissues were ground to a fine powder using a mixer mill as indicated above. Total RNA was extracted using the Spectrum Plant Total RNA Kit (Sigma-Aldrich, St. Louis, MO, USA) following the manufacturer’s protocol with the exception that 700 µL lysis buffer was used per seed. RNA (500 ng) was treated with DNase I (Amplification grade, Invitrogen, Carlsbad, CA, USA) to remove contaminating DNA. First-strand cDNA was synthesized using SuperScript II Reverse Transcriptase (Invitrogen). Parallel reactions were performed in the absence of Superscript II to test for genomic DNA contamination. Gene expressions from each cDNA sample were normalized to the endogenous reference PEPC16 [34]. The reactions (5 µL) consisted of 1 µL of first-strand cDNA (or untreated RNA) diluted 1/4 to 1/10, 250 nM of forward and reverse primers, and 2.5 μL of the iQ SYBR Green Supermix (BioRad, Hercules, CA, USA). qRT-PCR was performed on cDNA from four biological replicates or untreated RNA using a 7500 Realtime-PCR System (Applied Biosystems, Foster City, CA, USA). To verify the specificity of the qRT-PCR reactions, melting curves were determined subsequent to each reaction, and RT-PCR products for each primer set were fractionated on agarose gels. For the primer sequences, see Supplementary Table S2.

### 3.6. Degradation of Isoflavonoids

To obtain an extract that was concentrated in glyceollin I, or 6″-*O*-malonyldaidzin and daidzin, Harosoy 63 seeds (~500 g) were elicited for 48 h with 20 mg mL^−1^ *P. sojae* WGE, and the total isoflavonoids were extracted with 80% ethanol (2 µL mg^−1^ fresh tissue) using a Waring blender. Ethanol was removed by rotary evaporation and water by freeze-drying. The powder was reconstituted in 100 mL ethyl acetate and fractionated on ~20 preparative TLC plates according to [35]. The top band (*R*_f_ = 0.32) contained ~60% of glyceollin I and minor amounts of glyceollin II, III, coumestrol, and genistein, and was extracted separately from the origin containing 39% and 34% 6″-*O*-malonyldaidzin and daidzin, respectively. The extracts were resuspended in deionized water to an absorbance of 0.5–0.6 to be of physiologically relevant concentrations similar to that typically extracted from soybean seeds. The extract was sterilized by passage through nylon (0.22 μm). To test whether the seeds catalyzed the degradation of metabolites, seed coats were removed from surface-sterilized seeds that were imbibed overnight in water. The seeds were cut in half through the radical to separate the two cotyledons, and each cotyledon was incised once from the distal tip along the adaxial plane to ~3 mm from the radical. In 24-well plates in the dark, dissected half-seeds were incubated in 600 μL of sterile H_2_O or 5 mM AgNO_3_ for 2 h. These were then replaced with either extract enriched in glyceollin I, or 6″-*O*-malonyldaidzin and daidzin, or water, and incubated for 4 h. The seeds were extracted with 80% ethanol (1 µL mg^−1^ fresh tissue) as indicated above. The liquid surrounding the seeds was extracted three times with an equal volume of ethyl acetate. The pooled extracts were evaporated to dryness with nitrogen gas and reconstituted with the extract from the corresponding seed. Isoflaonoids were quantified from the extract by UPLC-PDA-MS^n^ as indicated above.

## 4. Conclusions

We have identified that combining the biotic elicitor WGE from *P. sojae* and the chemical elicitor AgNO_3_ stimulated the accumulation of the anticancer phytoalexin glyceollin I in an additive fashion, because they functioned by largely distinct elicitation mechanisms. WGE elicited a massive accumulation of biosynthesis gene mRNAs, and AgNO_3_ stimulated the hydrolysis of the isoflavone conjugate 6″-*O*-malonyldaidzin. Thus, our work suggests that overexpressing TFs that activate the transcription of phytoalexin biosynthesis genes and glycosidases that generate biosynthetic intermediates should be targets to genetically enhance biotic and chemical elicitation pathways, respectively. The combined elicitation approach represents an important discovery towards the economical bioproduction of glyceollin I and potentially other phytoalexins of medicinal or agricultural value.

## Supplementary Materials

Supplementary materials are available online.
