# Supplementary material for: Distinct Mechanisms of Biotic and Chemical Elicitors Enable Additive Elicitation of the Anticancer Phytoalexin Glyceollin I
Source: Molecules. 2017 Jul 27;22(8):1261. doi: 10.3390/molecules22081261 (PMC6152012; doi:10.3390/molecules22081261)
Supplement: Supplementary file 1 [file molecules-22-01261-s001.pdf]

Supplementary Figure S1

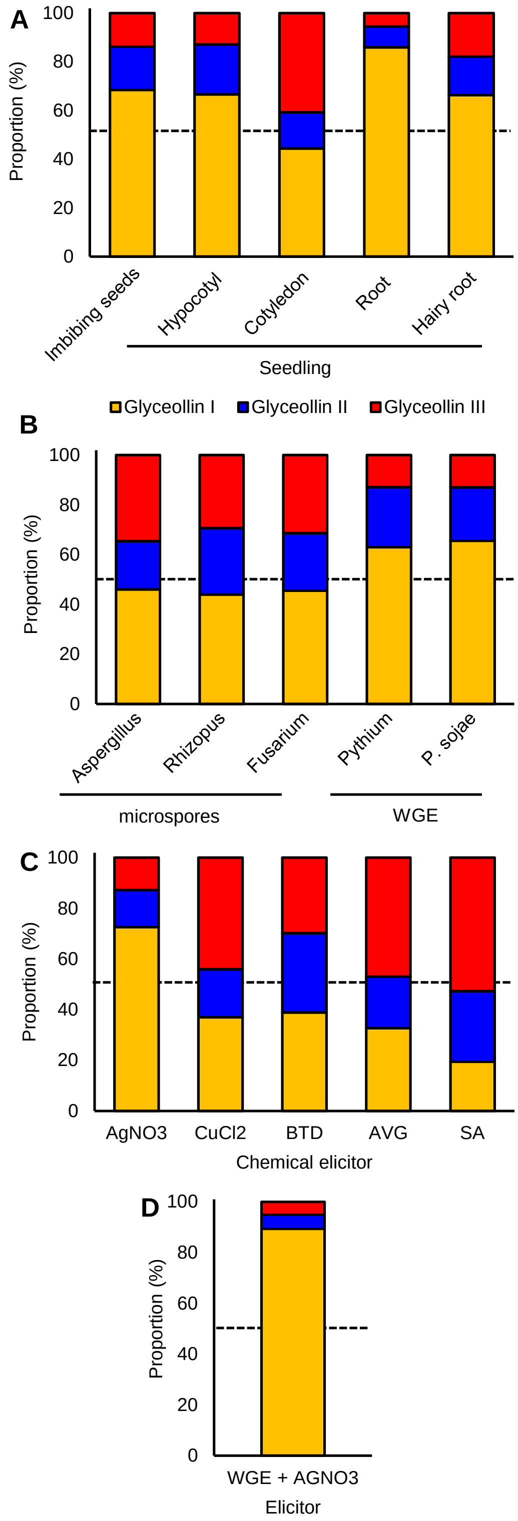

Supplementary Figure S1. Relative proportion of glyceollins from soybean organs treated with purified wall glucan elicitor (WGE) from *P. sojae* **(A)**, from soybean seeds treated with biotic elicitors **(B)**, chemical elicitors silver nitrate ($AgNO_3$), copper chloride ($CuCl_2$), benzothiadiazole (BTD), aminoethoxyvinyl glycine (AVG), and salicylic acid (SA) at 1 mM **(C)**, seeds with $AgNO_3$ (5 mM) and WGE (20 mg mL^{-1}) **(D)**. Dashed line demarcates 50%.

Supplementary Figure S2

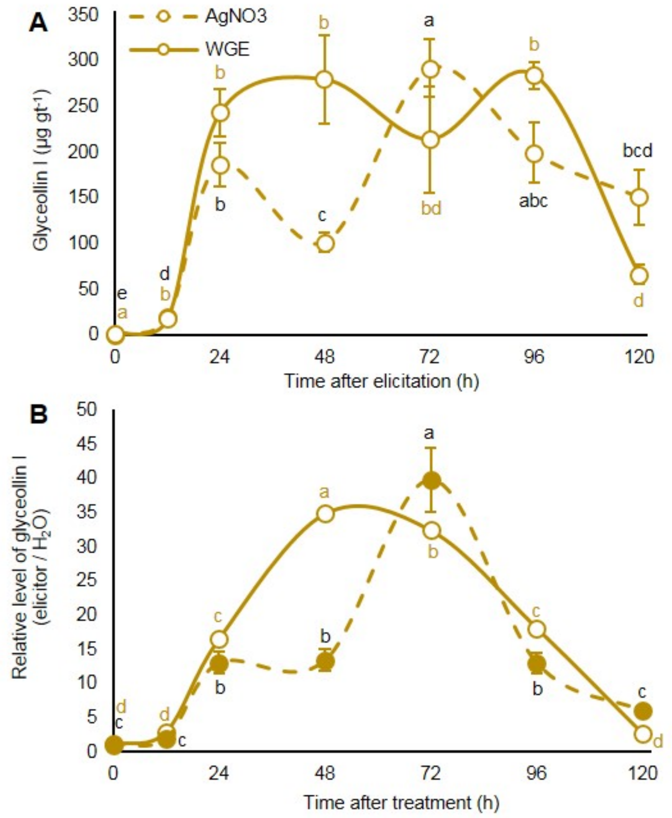

Supplementary Figure S2. (A) Glyceollin I accumulation dynamics after eliciting imbibed seeds with 5 mg mL^{-1} WGE or 2.5 mM $AgNO_3$ for the indicated times. **(B)** Metabolite induction compared to the solvent control (H_2O). Two-way ANOVA, Tukey post hoc test ($P < 0.001$).

Supplementary Figure S3

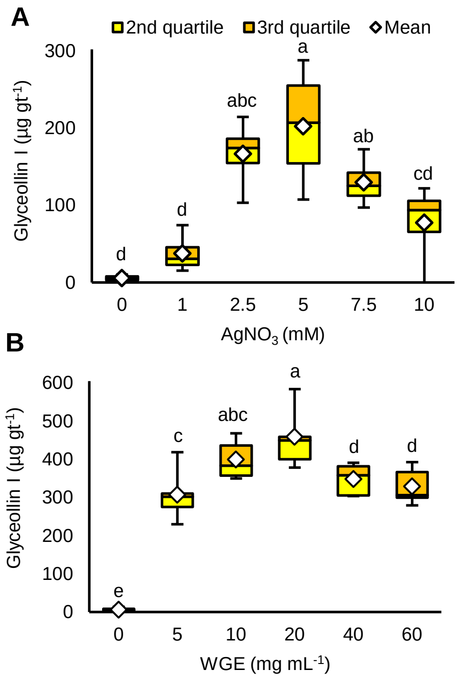

Supplementary Figure S3. (A) Glyceollin I concentrations in imbibing soybean seeds elicited with different concentrations of $AgNO_3$. **(B)** Elicitation with different concentrations of WGE. Two-way ANOVA, Tukey post hoc test, $P < 0.001$.

Supplementary Figure S4

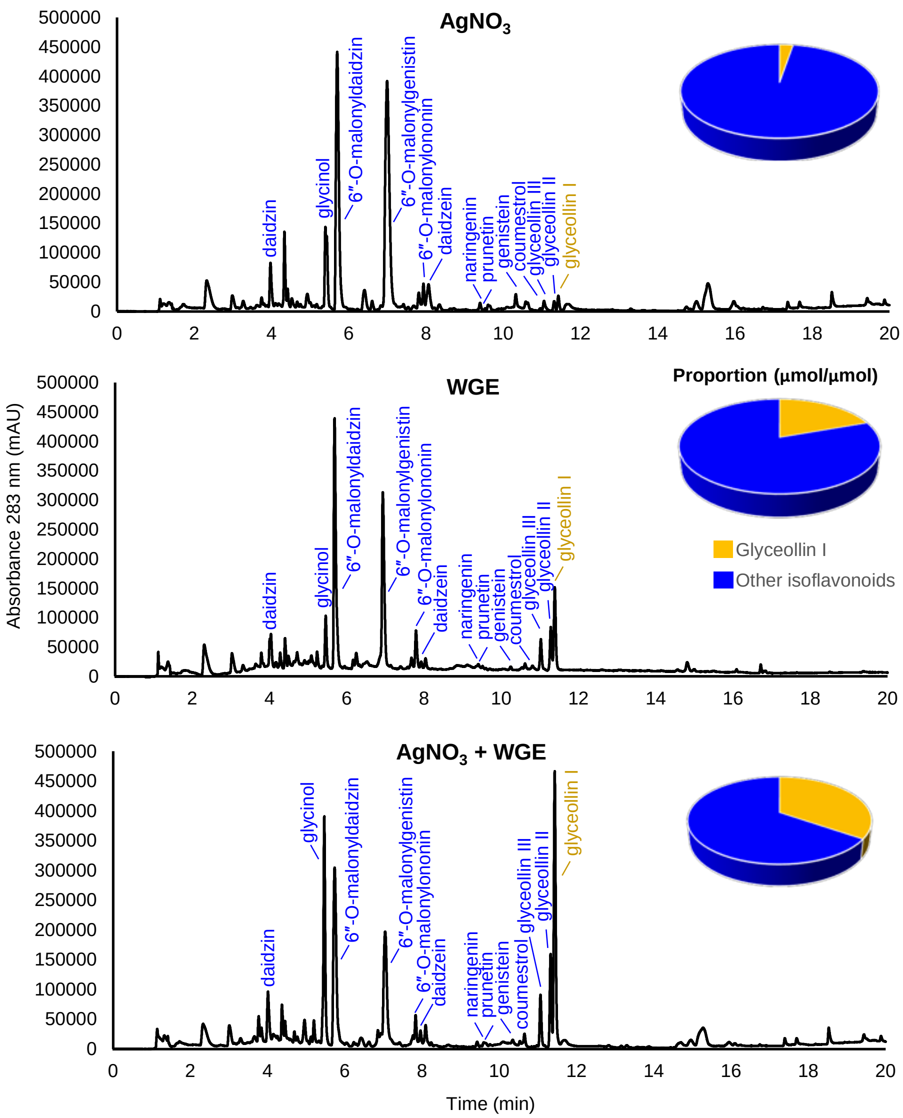

Supplementary Figure S4. Composition of glyceollin I relative to all other isoflavonoids in the seed ethanolic extract demonstated by UPLC-PDA and by pie chart. The amounts of all UPLC-PDA peaks that were not annotated were compounds for which we did not have standards and were quantified based on daidzin equivalents.

Supplementary Table S1. UPLC-PDA-MS^n identifying features of isoflavonoids.

Peak	R_t (min)	λ_{max} (nm)	$[M - H]^-$	MS/MS fragments$^-$ (*m*/z)	$[M + H]^+$	MS/MS fragments$^+$ (*m*/z)
daidzin[a]	4.39	250	415.10	253.05 [daidzein - H]$^-$	417.12	255.06 [daidzein + H]$^+$
glycinol[b]	5.47	283	271.06	217	255.07	227.59, 214.99
genistin[a]	5.48	260	431.10	-	433.11	271.07 [genistein + H]$^+$, 255.07
6''-O-malonyldaidzin[b]	5.80	251	501.10	253.05 [daidzein - H]$^-$	503.12	255.06 [daidzein + H]$^+$
6''-O-malonylgenistin[b]	7.01	260	517.10	269.05 [genistein - H]-	519.11	271.06 [genistein + H]$^+$
6''-O-malonylononin[b]	7.82	259	515.12	253.05 [daidzein - H]$^-$ 267.07 [formononetin - H]$^-$	517.13	269.08 [formononetin + H]$^+$
daidzein[a]	8.04	248	253.05	132.02	255.06	222.06
naringenin[a]	9.41	259	271.06	263.08	273.08	241.05
prunetin[a]	9.90	258	283.06	269.05 [genistein - H]-	285.08	-
genistein[a]	10.28	261	269.05	239.13	271.06	241.05
coumestrol[a]	10.90	342	267.03	253.09	269.04	236.05
glyceollin III[b]	11.10	289	337.11	319.10	339.12	321.11
glyceollin II[b]	11.30	283	337.11	319.10	339.12	321.11
glyceollin I[a]	11.48	283	337.11	255.07, 319.10	339.12	321.11

[a]Peak identities were based on MS feature comparisons to authentic standards.

[b]Identities based on MS feature comparisons to Aisyah et al. 2013 and Simons et al. 2014.

Supplementary Table S2. Primers.

Gene	Primer	Sequence
PEPC16	qPCf	TGCAACTGATTCTTATGTTCCAA
PEPC16	qPCr	GGCATCTTAACCACCTCACG
G4DT	qG4f	TGGCTTTCGGAGTTGGTATC
G4DT	qG4r	GAACAGCATTTCCCATACCC
IFS1	qIF1f	CACTCAAACTCGGGATCACA
IFS1	qIF1r	GACGCAAGTGCAGAAACAAA
IFS2	qIF2f	GGAGAGGTTGTTGAGGGTGA
IFS2	qIF2r	GACCCTTGATGTGGTCCTTG
I2’H	qI2f	CCATGCTTTTTGGTGGAACT
I2’H	qI2r	GCCTTCTTCAACACCTCTGG
